# Integrating MicroED into an X-ray Facility: Enjoying the Journey and Expanding Your Horizons

**DOI:** 10.1063/4.0000824

**Published:** 2025-10-27

**Authors:** Joseph D Ferrara

**Affiliations:** Rigaku Americas, The Woodlands, TX, USA

## Abstract

Installing a MicroED system involves several key considerations to ensure successful implementation. These include evaluating the site and addressing necessary infrastructure upgrades, such as HVAC, power, and water supply. Fortunately, MicroED instruments are designed to minimize these requirements, making the transition smoother. Additionally, modern software enables crystallographers to seamlessly apply their expertise from X-ray structure determination to MicroED experiments. In this presentation, we will explore the process of integrating MicroED into an existing X-ray facility, highlighting best practices and potential challenges.

